# MiR-23b controls ALDH1A1 expression in cervical cancer stem cells

**DOI:** 10.1186/s12885-017-3192-x

**Published:** 2017-04-27

**Authors:** Weiwen Wang, Yang Li, Na Liu, Yu Gao, Long Li

**Affiliations:** 1 Department of Gynecology and Obstetrics, First Affiliated Hospital, Xi’an Jiaotong University, 277 West Yanta Road, 710061, Xi’an, China; 2 Department of Neurosurgery, Tangdu Hospital, Fourth Military Medical University, Xi’an, China; 3 Department of Ultrasound, The Second Affiliated Hospital, Xi’an Jiaotong University, Xi’an, China

**Keywords:** miR-23b, Cervical cancer stem cells, ALDH1A1

## Abstract

**Background:**

Cancer stem cells has been widely investigated due to its essential role in cancer progression and drug resistance. Here, we try to find a new therapeutic target for cervical cancer stem cells.

**Methods:**

We detected ALDH1A1-associated miRNAs expression in our isolated tumorspheres and their corresponding parental cells. Sphere formation assay was also used to determine stemness after cells were manipulated with miR-23b plasmid or miR-23b inhibitor.

**Results:**

We found that miR-23b was under-expressed in cervical cancer stem cells to maintain high levels of ALDH1A1. Introduction of miR-23b into cervical cancer cells could alter stemness and cisplatin sensitivity.

**Conclusions:**

miR-23b plays key role in maintaining stemness of cervical cancer stem cells and can be developed as therapeutic target to better fight against cervical cancer.

**Electronic supplementary material:**

The online version of this article (doi:10.1186/s12885-017-3192-x) contains supplementary material, which is available to authorized users.

## Background

Cervical cancer is the major leading cause of cancer-related death in women worldwide. Annually, about 530,000 new cases of cervical cancer are documented, which puts people’s health into threat [1, 2]. Clinical and experimental evidence suggest that the infection of HPV is highly associated with the initiation and progression of cervical cancer [3, 4]. HPV-induced overexpression of E6 and E7 is the oncogenic stimulus to drive cervical cancer progression [1]. E6 forms complex with p53 and targets it via proteasome-mediated degradation, while E7 interacts and inactivates pRb, leading to the release of transcription factor E2F [5, 6]. Besides, other signaling pathways such as MAPK, PI3K, EGFR cassette are also involved in cervical carcinogenesis [1, 7–9]. Although standard treatments such as chemotherapy, radiotherapy and surgery have shown promising effect [10, 11], advanced tumor is observed in approximate 50% of patients. The increased population of cancer stem cells is one of mechanisms that promotes cervical cancer to advanced stage.

Cancer stem cells (CSCs) are small population that can self-renew and differentiate into cancerous cells if necessary [12, 13]. Just like other CSCs, Cervical cancer stem cells (CCSCs) are blinded to chemotherapy or radiation [14]. Evidence suggest that the recurrence of cervical cancer after remission is attributable to the failure of targeting CCSCs. As an intracellular enzyme that has a detoxifying role, ALDH1A1 has been identified as cancer stem cells marker for cervical cancer [15]. However, the detailed mechanisms responsible for higher expression of ALDH1A1 in CCSCs are largely unknown.

MiRNAs are short non-coding RNA molecules, usually 21–25 in length, which are processed from pri-miRNA and pre-miRNA by Drosha and Dicer, respectively [16]. The major biological function of miRNA is to titrate mRNA by directly binding its 3’UTR, leading to its degradation or translational suppression [16]. Growing evidence suggest that miRNAs are implicated into the pathogenesis of human diseases or cancers, including cervical cancer [17]. For instance, miR-150 promotes cervical cancer proliferation via degrading FOXO4 while miR-302 suppresses its growth by reducing AKT1 levels [18, 19].

In this study, we found that miR-23b was down-regulated in Hela and CaSki cervical cancer stem cells derived from tumorspheres. MiR-23b directly binds to the 3’UTR of ALDH1A1 to suppress its translation. Importantly, targeted expression of miR-23b could disrupt the stemness of CCSCs while miR-23b inhibitor increased the population of cervical tumorspheres. Meanwhile, miR-23b could alter the sensitivity of cervical cells to cisplatin treatment. Collectively, our data indicate that miR-23b may serve as cancer stem cell marker form cervical cancer and could be developed as therapeutic target.

## Methods

### Cell culture

Hela, CaSki, Siha and C33A cells were purchased from the American Type Culture Collection (ATCC, Manassas, VA, USA) and maintained in Dulbecco’s Modified Eagle’s Medium (Invitrogen, Grand Island, NY, USA) supplemented with 10% FBS, penicillin (100 units/ml), streptomycin (100 μg/ml), 1% L-glutamine. All cell lines were cultured in a 5% CO2 humidified incubator at 37 °C.

### Western blotting

Cells were lysed in RIPA buffer (50 mM Tris–HCl/pH 7.4, 1% NP-40, 150 mM NaCl, 1 mM EDTA, 1 mM PMSF, 1 mM Na3VO4, 1 mM NaF, 1 mM okadaic acid and 1 mg/ml aprotinin, leupeptin and pepstatin). Equal amount of proteins was loaded and separated on 8–10% SDS/PAGE gel and then transferred onto PVDF membranes (Millipore, Billerica, MA). After blocking in 5%BSA, the membranes were incubated with appropriate dilutions of specific primary antibodies overnight at 4 °C, followed by incubation of secondary antibody for 1 h at room temperature. Antibodies used in this study were: ALDH1A1 (Ab52492, Abcam), GAPDH (8C2, Santa Cruz).

### Luciferase assay

PLKO-miR-23b or PLKO bearing Hela cells were transfected with pGL3–3’UTR-ALDH1A1 or its mutated one (100 ng/well) using the Lipofectamine 3000 transfection reagent (Invitrogen, Carlsbad, CA), pRL-TK (10 ng/well) was used as transfection control. After 24 h transfection, cells were lysed for Dual-Luciferase Reporter Assay detection (Promega, Madison, WI)) according to the manufacturer’s protocol.

### Site directed mutagenesis

Briefly, PCR was conducted in 20 μL volume system: 50 ng DNA template (pGL3–3’UTR-ALDH1A1), 2 μL 10XPhusion buffer, 0.25 μL Phusion enzyme, 1 μL 10 μM forward or reverse primer. The reaction was done as follows: 98 °C 5 mins, 30 cycles of: 98 °C 30s, 60 °C 30s, 72 °C 2 mins. PCR products was digested with Dpn I and subjected to PNK treatment, then was ligated with T4 ligase before transformation. Mutated clone was confirmed by Hind III restriction enzyme. The primers used in mutagenesis is: forward, 5′-TTGCAAACCCCCAAGTCCTATCCTA;

reverse, 5′-TCAGAAGGCAAATAATTCTTTCAG.

### RNA isolation and real time PCR

total RNAs were isolated using Trizol reagent (Invitrogen, Grand Island, NY). 1 μg of total RNA was subjected to reverse transcription using Superscript III transcriptase (Invitrogen, Grand Island, NY). Quantitative real-time PCR (qRT-PCR) was conducted using a Bio-Rad CFX96 system with SYBR green to determine the mRNA expression level of a gene of interest. The reverse transcription of MiRNAs was performed: 1) poly A addition: 1 unit of Poly (A) polymerase with 1 mM ATP in 1xRT buffer at 37 °C for 10 min in 10 μl volume, and then heat inactivate at 95 °C for 2 min, 2) Primer anchor: add 50 mmol anchor primer to 12.5 μl, incubate at 65 °C for 5 min, 3) cDNA synthesis: add 2 μl 5× RT buffer, 2 μl 10 mM dNTP, 1 μl reverse transcriptase to total 20 μl, incubate at 42 °C for 1 h. Quantitative real-time PCR (qRT-PCR) of miRNA was conducted using the Taqman probe. U6 was served as control.

### Sphere formation assay

5 × 10^3^ cells were mixed with equal amount of growth factor enriched matrigel. 100 μL mixture was seeded into 24-well plate and supplemented with 1 mL medium. After 2 weeks later, floating cells were counted under microscopic machine.

### Statistics

Differences in mean values between two groups were analyzed by two-tailed Student’s t test and the data values were presented as the mean ± SEM. *p* ≤ 0.05 was considered as statistical significance.

## Results

### MiR-23b is down-regulated in cervical cancer stem cells

**Fig. 1:**
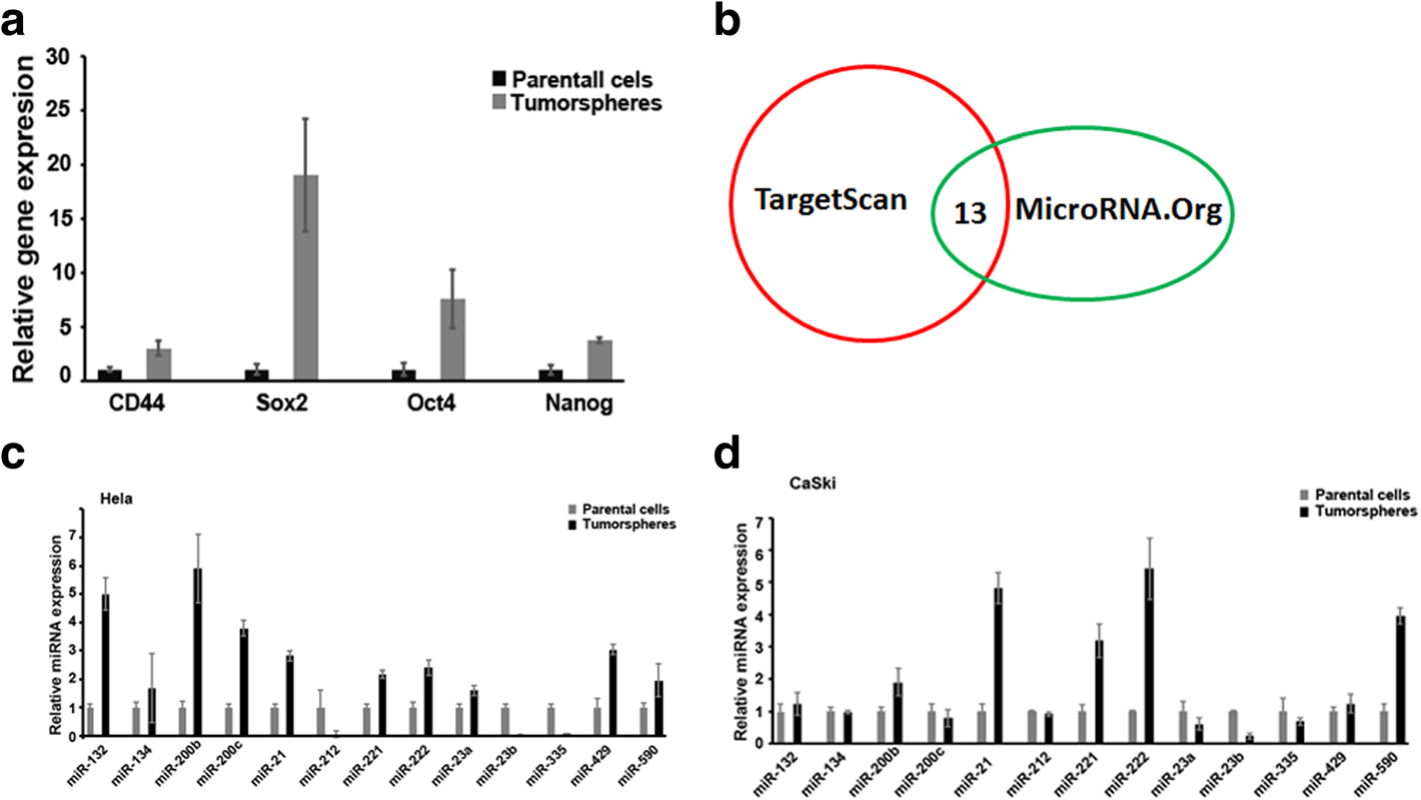
MiR-23b was down-regulated in sphere-forming cells. **a**. confirmation of sphere-forming cells as cancer stem cells by using several markers: Nanog, Oct4, CD44 and Sox2. Genes were normalized to GAPDH. **b**. TargetScan and MicroRNA.Org were performed to predict miRNAs for ALDH1A1. **c**. The expression of predicted miRNAs between tumorsphere cells and parental cells in Hela. **d**. The predicted miRNAs (**c**) in between tumorsphere cells and parental cells in CaSki. Data is presented as mean ± SEM. *P < 0.05; **P < 0.01; ***P < 0.001

ALDH1A1 has been proved as one marker for cervical cancer stem cells. We hypothesized that the higher levels of ALDH1A1 in cervical cancer stem cells are partially attributable to the deregulation of miRNAs. For this end, we isolated miRNAs from sphere- forming cells, which were verified by stem cell markers: Nanog, Oct4, CD44 and Sox2 (Fig. 1a), and compared their expression levels to the corresponding partners in parental cells. Using two miRNA prediction programs, we finally focused on 13 miRNAs because they are predicted by both programs to target ALDH1A1 (Fig. 1b). Results showed that miR-23b was consistently down-regulated in sphere-forming cells derived from four cervical cancer cells (Fig. 1c, d and Additional file 1: Figure S1), suggesting that it may be good candidate for targeting ALDH1A1.

### MiR-23b directly binds to the 3’UTR of ALDH1A1

**Fig. 2:**
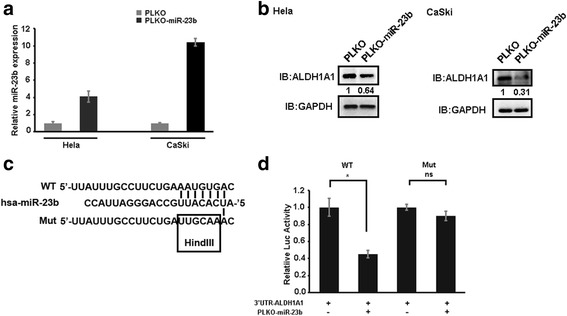
MiR-23b directly suppresses ALDH1A1 expression via base-pairing to its 3’UTR. **a**. Overexpression efficiency of miR-23b in Hela and CaSki cells. **b**. ALDH1A1 was suppressed by overexpressing miR-23b in Hela and CaSki cells. GAPDH was used as loading control. **c**. Schematic map of miR-23b binding seed on 3’UTR of ALDH1A1. **d**. 3’UTR binding assay to confirm miR-23b directly interacts with 3’UTR of ALDH1A1. Data is presented as mean ± SEM. *P < 0.05; **P < 0.01; ***P < 0.001

To test whether miR-23b could decrease ALDH1A1 expression levels. We overexpressed miR-23b in Hela cells and CaSki cells (Fig. 2a), finding that miR-23b indeed bears the ability to reduce ALDH1A1 expression levels in both Hela and CaSki cell lines (Fig. 2b). To examine whether miR-23b exerts its degradation effect on ALDH1A1 via binding to its 3’UTR, we cloned luciferase-based 3’UTR of ALDH1A1 and found its activity can be reduced by miR-23b (Fig. 2c, d). Importantly, this reduced luciferase activity can be blocked when the predicted binding site is mutated (Fig. 2c, d), suggesting that miR-23b directly bind to the 3’UTR of ALDH1A1 to cause its reduction.

### MiR-23b could alter stemness of cervical cancer stem cells

**Fig. 3:**
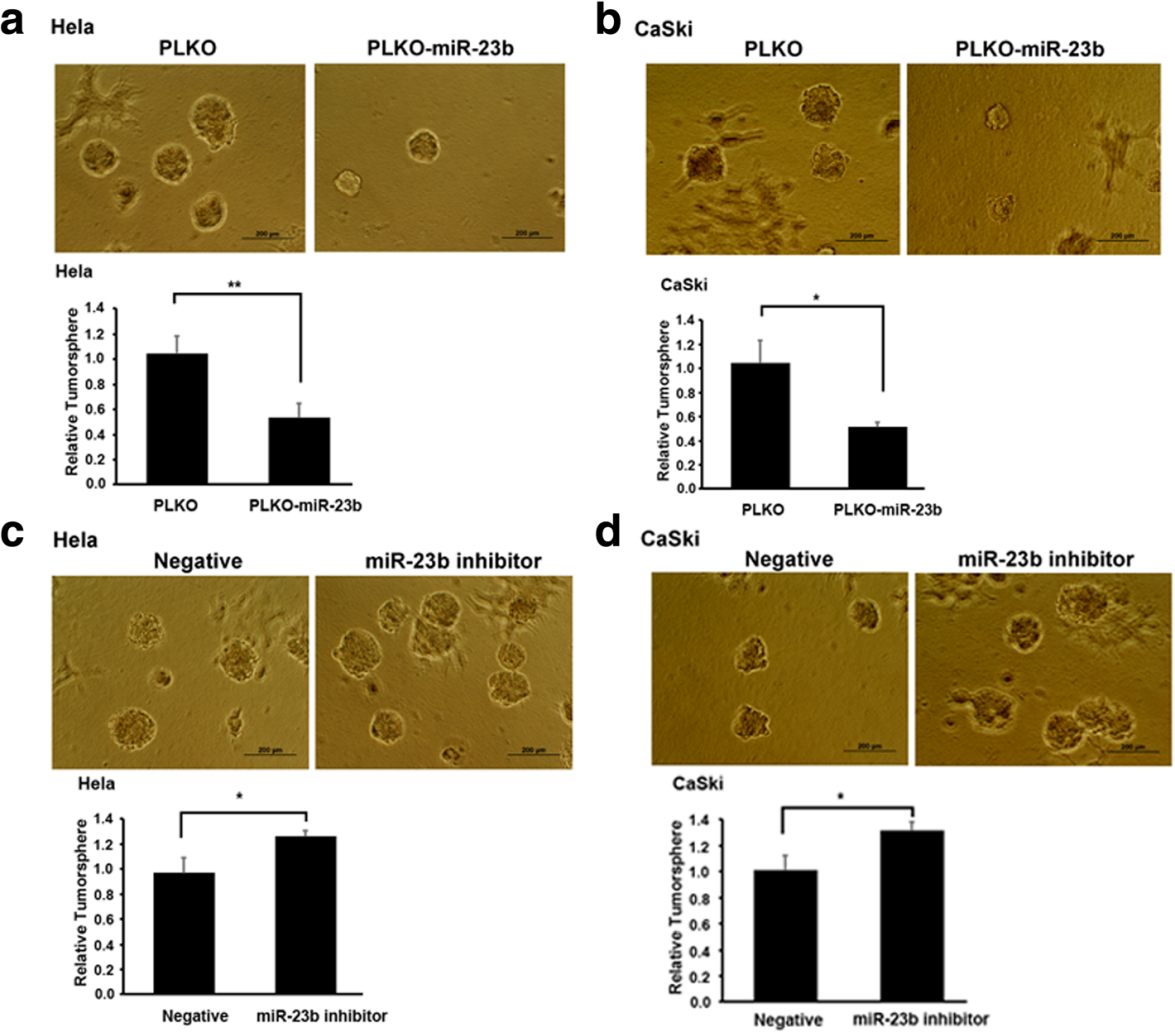
MiR-23b could alter the stemness of cervical cancer stem cells. **a**-**b**. Forced expression into Hela cells **a** and CaSki cells **b** reduced the number of tumorspheres. **c-d** Addition of miR-23b inhibitor into Hela cells **c** and CaSki cells **d** increased the number of tumorspheres. Data is presented as mean ± SEM. *P < 0.05; **P < 0.01; ***P < 0.001

Since ALDH1A1 plays key role in maintaining the homeostasis of cervical cancer stem cell, we wanted to detect the sphere-forming ability of cells which were transfected with miR-23b. As shown in Fig. 3a, overexpression of miR-23b in Hela cells dramatically reduce the size and number of tumorspheres. Similar result was gained when Hela cells were replaced by CaSki cells (Fig. 3b).

On the contrary, introduction of miR-23b inhibitor into Hela cells bestowed them strong capacity of forming tumorspheres (Fig. 3c). We also observed similar phenomenon in CaSki cells which were transfected with miR-23b inhibitor (Fig. 3d).

Together, these data suggest that miR-23b could change the stemness of cervical cancer stem cells, probably via inhibiting the expression of ALDH1A1.

### MiR-23b re-sensitizes cervical cancer cells to cisplatin treatment

**Fig. 4:**
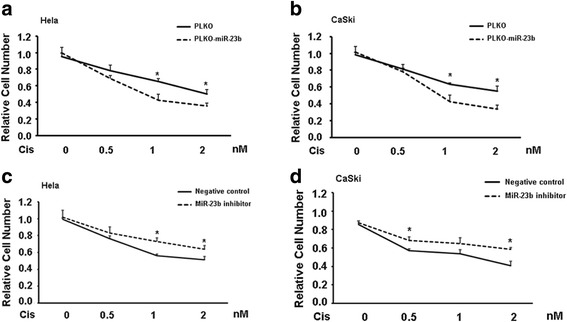
MiR-23b confers cisplatin sensitivity to cervical cancer cells. **a**-**b**. Introduction of miR-23b into Hela cells **a** and CaSki cells **b** sensitized cells to cisplatin treatment. **c**-**d**. Addition of miR-23b inhibitor into Hela cells **c** and CaSki cells **d** makes cells more resistant to cisplatin treatment. Data is presented as mean ± SEM. *P < 0.05; **P < 0.01; ***P < 0.001

Cisplatin treatment is one of options for advanced cervical cancer [20, 21]. Numerous studies have proved that cancer stem cells contribute to drug resistance including cisplatin resistance [22]. To test whether miR-23b meditated stemness of cervical cancer stem cells is implicated into cisplatin sensitivity, we administrated various concentrations of cisplatin into vector-bearing and miR-23b overexpressing Hela cells. Result demonstrated that miR-23b could re-sensitize Hela cells to cisplatin treatment (Fig. 4a). Similar result was obtained in CaSki cells (Fig. 4b).

On the contrary, miR-23b inhibitor could confer cisplatin resistance to both Hela and CaSki cells (Fig. 4c, d). All these data suggest that miR-23b alters cisplatin sensitivity by affecting the population of cervical cancer stem cells.

## Discussion

Cancer stem cells confer drug resistance to patients who received conventional treatments such as chemotherapy and radiotherapy. Here, by screening 13 miRNAs that could predicatively bind the 3’UTR of ALDH1A1 for RISC-mediated degradation, we found that miR-23b accordantly exhibits lower expression in sphere cells derived from four cervical cancer cell lines (Hela, CaSki, C33A and Siha), indicating the downregulation of miR-23b is generally observed in cervical cancer stem cells (CCSCs). Functionally, forced expression of miR-23b could disrupt cancer stemness via suppressing ALDH1A1 expression while addition of miR-23b inhibitor bore the ability to increase cancer stem cells population. 3’UTR binding assay also confirmed that miR-23b indeed directly binds to ALDH1A1 mRNA, which could be attenuated by destroying the binding motif. Importantly, miR-23b mediated reduction of cervical cancer stem cells also contributed to cisplatin sensitivity.

According to our data, miR-23b plays role in altering cell sensitivity to cisplatin treatment, probably due to the consequence of the changed population of CCSCs. Since CCSCs are inherently blinded to chemotherapy or radiation, we believe that miR-23b still can alter radiation sensitivity of cervical cancer cells, which requires future investigation. In addition, it is appealing to see whether the expression levels of miR-23b is silenced in cisplatin resistant cervical cancer cells (Cis-res CCCs) and whether addition of miR-23b mimic could re-sensitizes Cis-res CCCs to cisplatin treatment.

Serval reports have demonstrated that miR-23b is down-regulated in HPV-induced cervical cancer and this regulation is dependent of p53, which can bind to the promoter of miR-23b and trans-activate miR-23b expression [23]. As we known, p53 undergoes degradation upon HPV infection. Of note, p53 is inactivated in CCSCs for the maintenance of stemness. We postulate that under-expression of miR-23b in CCSCs is regulated at least partially by inactivation of p53. Additionally, one study has pointed that the promoter of miR-23b is the potential methylation target [24]. Therefore, the further suppressed expression of miR-23b in CCSCs is possibly regulated by methylation modification. MiR-23b has been proved as tumor suppressive miRNA in many cancers. In gastric carcinoma, forced expression of miR-23b inhibits proliferation, invasion, epithelial-mesenchymal transition (EMT) and tumorsphere formation of gastric cancer cells via targeting Notch2 receptor [25]. MiR-23b also can target cyclin G1 to suppress ovarian cancer progression [26]. Finally, prostate cancer tumorigenesis is highly associated with miR-23b levels, which base-pairs with 3’UTR of Src to inhibit its translation [27]. Here, we first showed that ALDH1A1 is additional target for miR-23b, indicating that miR-23b exerts its functions via affecting numerous genes’ expression. Of note, miR-23b has been shown to be downregulated in cervical cancer cells due to the hypermethylation at its promoter locus. To supplement previous finding, we found that miR-23b is further reduced in isolated tumorspheres, for the purpose of maintaining ALDH1A1 levels and cells’ stemness. Therefore, developing miR-23b as therapeutic target will better battle against cervical cancer.

## Conclusion

We found that miR-23b is under-expressed in sphere cells compared to the parental cells, which contributes to the higher expression levels of ALDH1A1 in sphere cells. Forced expression of miR-23b has capacity to reduce the number of tumorspheres, re-sensitizing cells to cisplatin treatment. All data suggest that there is need to develop miR-23b as therapeutic target for cervical cancer stem cells.

## Additional file

Additional file 1: Figure S1. MiR-23b is under-expressed in tumorsphere cells derived from Siha and C33A cells. Data is presented as mean ± SEM. **P < 0.05; **P < 0.01*. (TIFF 613 kb)

## Abbreviations

CSC: Cancer stem cell
CCSC: Cervical cancer stem cell

## References

[1] Manzo-Merino J, Contreras-Paredes A, Vazquez-Ulloa E, Rocha-Zavaleta L, Fuentes-Gonzalez AM, Lizano M (2014). The role of signaling pathways in cervical cancer and molecular therapeutic targets. Arch Med Res.

[2] Siegel RL, Miller KD, Jemal A (2016). Cancer statistics, 2016. CA Cancer J Clin.

[3] Thomas M, Narayan N, Pim D, Tomaic V, Massimi P, Nagasaka K, Kranjec C, Gammoh N, Banks L (2008). Human papillomaviruses, cervical cancer and cell polarity. Oncogene.

[4] Yim EK, Park JS (2005). The role of HPV E6 and E7 oncoproteins in HPV-associated cervical carcinogenesis. Breast Cancer Res Treat.

[5] Thomas M, Pim D, Banks L (1999). The role of the E6-p53 interaction in the molecular pathogenesis of HPV. Oncogene.

[6] Bernard X, Robinson P, Nomine Y, Masson M, Charbonnier S, Ramirez-Ramos JR, Deryckere F, Trave G, Orfanoudakis G (2011). Proteasomal degradation of p53 by human papillomavirus E6 oncoprotein relies on the structural integrity of p53 core domain. Plos One.

[7] Wu J, Chen C, Zhao KN (2013). Phosphatidylinositol 3-kinase signaling as a therapeutic target for cervical cancer. Curr Cancer Drug Targets.

[8] Branca M, Ciotti M, Santini D, Bonito LD, Benedetto A, Giorgi C, Paba P, Favalli C, Costa S, Agarossi A (2004). Activation of the ERK/MAP kinase pathway in cervical intraepithelial neoplasia is related to grade of the lesion but not to high-risk human papillomavirus, virus clearance, or prognosis in cervical cancer. Am J Clin Pathol.

[9] Bumrungthai S, Munjal K, Nandekar S, Cooper K, Ekalaksananan T, Pientong C, Evans MF (2015). Epidermal growth factor receptor pathway mutation and expression profiles in cervical squamous cell carcinoma: therapeutic implications. J Transl Med.

[10] Glick SB, Clarke AR, Blanchard A, Whitaker AK (2012). Cervical cancer screening, diagnosis and treatment interventions for racial and ethnic minorities: a systematic review. J Gen Intern Med.

[11] Schreuder SM, Lensing R, Stoker J, Bipat S (2015). Monitoring treatment response in patients undergoing chemoradiotherapy for locally advanced uterine cervical cancer by additional diffusion-weighted imaging: A systematic review. J Magn Reson Imaging.

[12] Kreso A, Dick JE (2014). Evolution of the cancer stem cell model. Cell Stem Cell.

[13] Nguyen LV, Vanner R, Dirks P, Eaves CJ (2012). Cancer stem cells: an evolving concept. Nat Rev Cancer.

[14] Chhabra R (2015). Cervical cancer stem cells: opportunities and challenges. J Cancer Res Clin Oncol.

[15] Douville J, Beaulieu R, Balicki D (2009). ALDH1 as a functional marker of cancer stem and progenitor cells. Stem Cells Dev.

[16] Ha M, Kim VN (2014). Regulation of microRNA biogenesis. Nat Rev Mol Cell Biol.

[17] Banno K, Iida M, Yanokura M, Kisu I, Iwata T, Tominaga E, Tanaka K, Aoki D (2014). MicroRNA in cervical cancer: OncomiRs and tumor suppressor miRs in diagnosis and treatment. Sci World J.

[18] Li J, Hu L, Tian C, Lu F, Wu J, Liu L (2015). microRNA-150 promotes cervical cancer cell growth and survival by targeting FOXO4. BMC Mol Biol.

[19] Cai N, Wang YD, Zheng PS (2013). The microRNA-302-367 cluster suppresses the proliferation of cervical carcinoma cells through the novel target AKT1. RNA.

[20] Tan S, Hougardy BM, Meersma GJ, Schaap B, de Vries EG, van der Zee AG, de Jong S (2012). Human papilloma virus 16 E6 RNA interference enhances cisplatin and death receptor-mediated apoptosis in human cervical carcinoma cells. Mol Pharmacol.

[21] Sahin K, Tuzcu M, Basak N, Caglayan B, Kilic U, Sahin F, Kucuk O (2012). Sensitization of Cervical Cancer Cells to Cisplatin by Genistein: the Role of NFkappaB and Akt/mTOR Signaling Pathways. J Oncol.

[22] Shen DW, Pouliot LM, Hall MD, Gottesman MM (2012). Cisplatin resistance: a cellular self-defense mechanism resulting from multiple epigenetic and genetic changes. Pharmacol Rev.

[23] Au Yeung CL, Tsang TY, Yau PL, Kwok TT (2011). Human papillomavirus type 16 E6 induces cervical cancer cell migration through the p53/microRNA-23b/urokinase-type plasminogen activator pathway. Oncogene.

[24] Campos-Viguri GE, Jimenez-Wences H, Peralta-Zaragoza O, Torres-Altamirano G, Soto-Flores DG, Hernandez-Sotelo D, Alarcon-Romero Ldel C, Jimenez-Lopez MA, Illades-Aguiar B, Fernandez-Tilapa G (2015). miR-23b as a potential tumor suppressor and its regulation by DNA methylation in cervical cancer. Infect Agents Cancer.

[25] Huang TT, Ping YH, Wang AM, Ke CC, Fang WL, Huang KH, Lee HC, Chi CW, Yeh TS (2015). The reciprocal regulation loop of Notch2 pathway and miR-23b in controlling gastric carcinogenesis. Oncotarget.

[26] Yan J, Jiang JY, Meng XN, Xiu YL, Zong ZH (2016). MiR-23b targets cyclin G1 and suppresses ovarian cancer tumorigenesis and progression. J Exp Clin Cancer Res.

[27] Majid S, Dar AA, Saini S, Arora S, Shahryari V, Zaman MS, Chang I, Yamamura S, Tanaka Y, Deng G (2012). miR-23b represses proto-oncogene Src kinase and functions as methylation-silenced tumor suppressor with diagnostic and prognostic significance in prostate cancer. Cancer Res.

