# Supplementary figures and images for: MiR-23b controls ALDH1A1 expression in cervical cancer stem cells
Source: BMC Cancer. 2017 Apr 27;17:292. doi: 10.1186/s12885-017-3192-x (PMC5408421; doi:10.1186/s12885-017-3192-x)

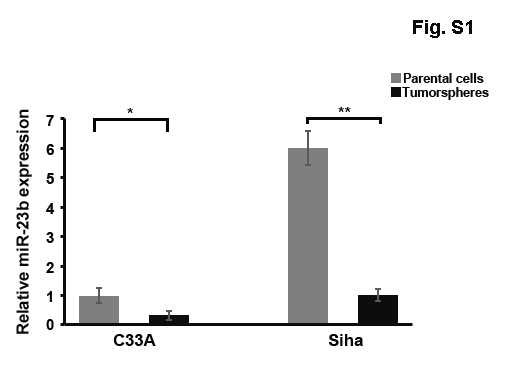

Supplement: Additional file 1: Figure S1. — MiR-23b is under-expressed in tumorsphere cells derived from Siha and C33A cells. Data is presented as mean ± SEM. *P < 0.05; **P < 0.01. (TIFF 613 kb) [file 12885_2017_3192_MOESM1_ESM.tif]
